# *In vitro* Effects of Biologically Active Vitamin D on Myogenesis: A Systematic Review

**DOI:** 10.3389/fphys.2021.736708

**Published:** 2021-09-09

**Authors:** Kathryn H. Alliband, Sofia V. Kozhevnikova, Tim Parr, Preeti H. Jethwa, John M. Brameld

**Affiliations:** Division of Food Nutrition and Dietetics, School of Biosciences, University of Nottingham Sutton Bonington Campus, Loughborough, United Kingdom

**Keywords:** vitamin D, 25-dihydroxyvitamin D3, 25-hydroxyvitamin D2, myogenesis, myogenin, differentiation, MyoD, systematic review

## Abstract

Vitamin D (VD) deficiency is associated with muscle weakness. A reduction in the incidence of falls in the elderly following VD supplementation and identification of the VD receptor within muscle cells suggests a direct effect of VD on muscle, but little is known about the underlying mechanisms. Here we systematically searched the literature to identify effects of active VD [1,25(OH)2D3] on skeletal muscle myogenesis *in vitro*, with no restriction on year of publication. Eligibility was assessed by strict inclusion/exclusion criteria and agreed by two independent investigators. Twelve relevant pa-pers were identified using four different cell types (C2C12, primary mouse satellite cells, primary chick myoblasts, and primary human myoblasts) and a range of myogenic markers (myoD, myogenin, creatine kinase, myosin heavy chain, and myotube size). A clear inhibitory effect of 1,25(OH)2D3 on proliferation was reported, while the effects on the different stages of differentiation were less consistent probably due to variation in cell type, time points and doses of 1,25(OH)2D3 used. However, myotube size was consistently increased by 1,25(OH)2D3. Overall, the evidence suggests that 1,25(OH)2D3 inhibits proliferation and promotes differentiation of myoblasts, but future studies should use time courses to gain a clearer understanding.

## Introduction

The link between vitamin D (VD) and bone health has been studied extensively, but recent evidence points toward a relationship between VD and skeletal muscle function (Wiciński et al., 2019). Muscle biopsies from VD deficient individuals show muscle wasting (mostly type II fibre atrophy), large interfibrillar spaces, and fat infiltration within the muscle (Ceglia, 2008). In general, deficiency occurs when levels of 25(OH)D3 (inactive vitamin D) fall below 25 nmol/L, however this cut-off point can vary within the literature (Halfon et al., 2015). In the elderly population, vitamin D deficiency has been linked to an increased risk of falls which is thought to be partly due to muscle weakness and wasting (Garcia et al., 2011).

**Figure 1:**
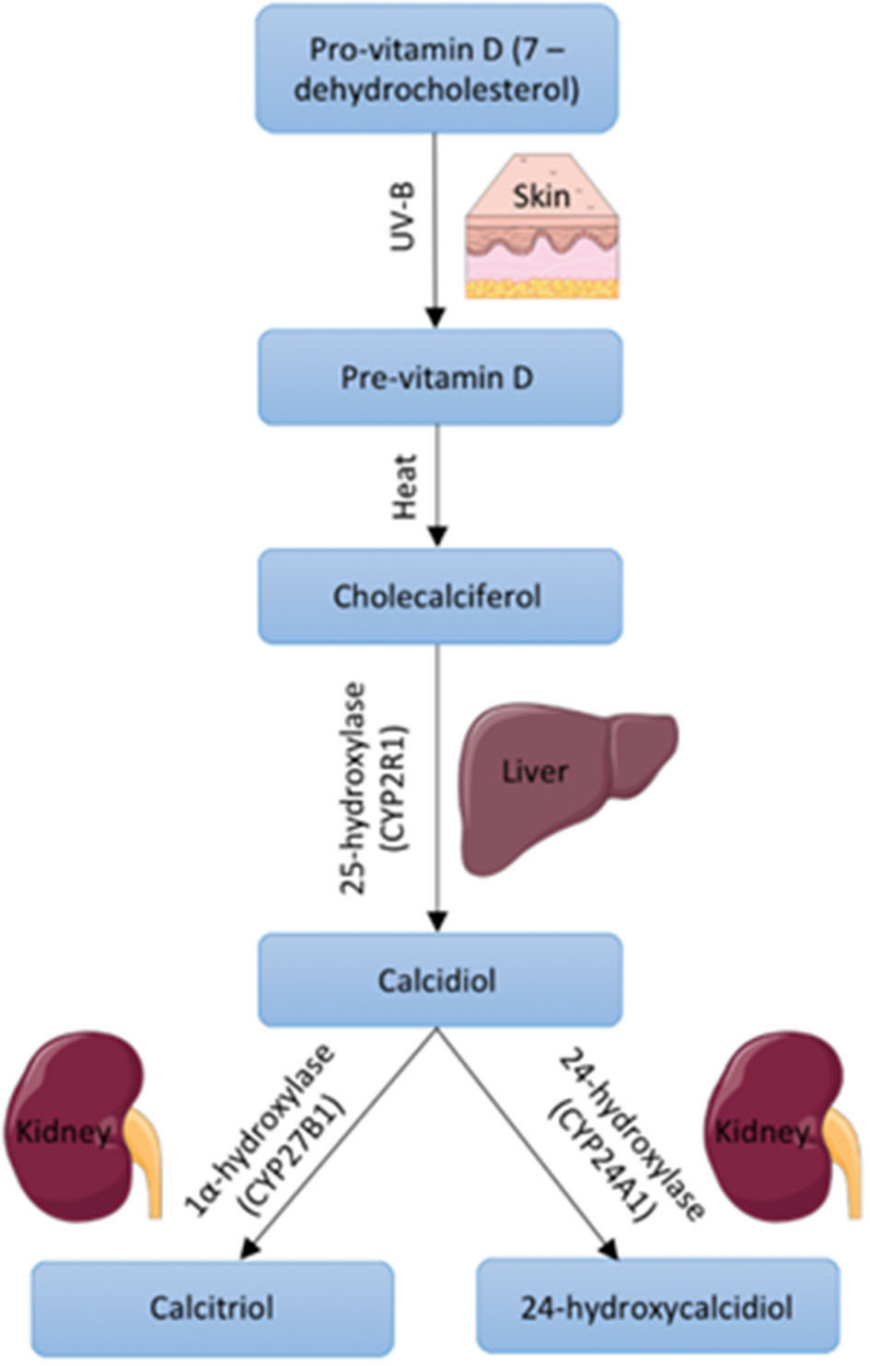
Vitamin D synthesis within the body including precursors, enzymes, and body site. Images used within this figure were obtained from smart servier medical art and can be found at https://smart.servier.com.

Around 80–90% of VD is obtained via UV-B induced synthesis in the skin in humans, whilst 10–20% comes from dietary intake (Halfon et al., 2015). In the skin, 7-dehydrocholesterol is converted to pre-vitamin D upon UV-B radiation. This is then converted to cholecalciferol which becomes bound to VD binding globulin and this complex is transported to the liver where it undergoes hydroxylation by 25-hydroxylase to form 25(OH)D3 or calcidiol (Hamilton, 2010). 25(OH)D3 is the major circulating form of VD and is measured as a marker of VD status (Pojednic and Ceglia, 2014). A final step, to produce the biologically active form of VD, involves hydroxylation by 1α-hydroxylase to produce 1,25(OH)2D3 otherwise known as calcitriol (Hamilton, 2010; Figure 1). 1α-hydroxylase is expressed largely in the kidney, which contributes to active VD in the circulation, however the enzyme is also expressed within other tissues such as muscle, which allows local conversion of inactive to active VD (Ceglia, 2008).

Studies in both chicken and human skeletal muscle have identified the presence of the vitamin D receptor (VDR) within muscle cells thereby providing evidence for a direct effect of VD on muscle (Zanello et al., 1997; Bischoff et al., 2001). This has since been supported by human studies which have found that low serum 25(OH)D3 concentrations in elderly individuals is associated with reduced muscle strength and an increased risk of falls (Ceglia and Harris, 2013). These effects of VD deficiency on muscle appear to be reversible with supplementation in the elderly population leading to beneficial outcomes such as increased strength, balance, and a decreased risk of falls (Harwood et al., 2004). This effect is thought to be, at least in part, directly through the VDR present in muscle cells. VDR knockout mice have been found to have muscle fibres which are 20% smaller in size than controls as well as smaller body size, weight, and impaired motor co-ordination (Pojednic and Ceglia, 2014).

Within the literature, VDRs have been described in different cell locations, one as a nuclear hormone receptor and the other as a membrane receptor (Ceglia, 2008). The origin of the membrane receptor is unclear, some argue there is a distinct membrane receptor, however the majority of evidence points toward one VDR with the ability to translocate between the nucleus and membrane (Halfon et al., 2015).

It is well-known that the VDR has a nuclear hormone receptor function, with the transcription of over 900 genes found to be affected upon treatment with active VD (Wang et al., 2005). 1,25(OH)2D3, binds to the VDR which induces heterodimerisation with the retinoid X receptor (RXR). This complex is then able to bind to VD response elements (VDREs) to activate or repress transcription of target genes (Halfon et al., 2015). Expression of genes involved in myogenic proliferation and differentiation have been shown to change upon treatment with VD leading to the suggestion that VD may have a direct effect on myogenesis (Wiciński et al., 2019).

The aim of this systematic review is to summarise the current body of evidence on the effects of active VD on skeletal muscle cells in culture. There is conflicting evidence in this area, therefore this review aims to summarise, assess, and interpret the current body of evidence and identify areas where further investigation is required.

## Materials and Methods

This review was constructed in accordance with the Preferred Reporting Items for Systematic Reviews and Meta-Analyses (PRISMA) guidelines (Moher et al., 2009).

### Search and Selection Criteria

Relevant papers were identified through the computerised search databases (PubMed (MEDLINE), Web of Science and Google Scholar). The search process followed the population (P), Intervention (I), Comparison (C), and outcome (O, PICO). The review population was *in vitro* models of muscle cells, the intervention was active VD treatment, comparison was controls not treated with VD and the measuring outcomes were the effects of active VD on muscle proliferation and differentiation. Specific search terms “vitamin D OR 1,25Dihydroxyvitamin D3 OR 1,25(OH)2D3 OR calcitriol AND myogenesis OR muscle differentiation” were used to obtain relevant articles. To obtain the relevant articles, two independent reviewers (KHA & SVK) assessed the titles, abstract and full articles based on a strict inclusion and exclusion criteria and if any disagreements arose, these were resolved by discussion. Finally, the reference list of these were searched to find any additional papers.

### Selected Articles Criteria

Articles were not restricted to any dates as there have been no previous systematic reviews conducted investigating the literature relating to active VD and myogenesis *in vitro*.

Inclusion Criteria

- Studies must have been written in English to avoid any translation errors.
- All articles must have described an *in vitro* model of muscle cells (primary or cell line).
- Any form of active VD can be considered [1,25(OH)2D3 or active VD analogues].
- Treatment of VD must be of known quantity and administered alone and not in combination with other drugs/vitamins/minerals.
- Must determine effects on proliferation/differentiation of muscle cells.

Exclusion Criteria

- Whole animal or human models.
- Systematic reviews or critical reviews.
- Studies investigating VD receptor and not VD.
- Studies investigating cancer or ageing.

### Measured Outcomes

The primary measured outcomes of this review are markers of myogenesis such as level of DNA synthesis, mRNA and protein levels of myoD, myogenin, myosin/myosin heavy chain isoforms, creatine kinase activity, and myotube size. There were no secondary measured outcomes.

### Data Extraction

Using a standard extraction form, data from all studies were extracted and charted using Excel (Microsoft Excel, Washington, USA). Data extracted included title, author, publication year, muscle cell model used, exposure to VD, and outcomes (DNA, myogenin, myoD, creatine kinase, myosin, and myotube size).

All key characteristics of the selected papers were expressed in tables. These included the study design, model used, number of samples, outcome measures, and doses of VD converted to moles for consistency.

### Data Analysis

The significant effects (*p* < 0.05) in response to VD were charted to compare across the articles reviewed, however some values were read from graphs where raw data was not provided so are best estimates. Changes in expression were used to generate bar graphs using Excel (Microsoft Excel, Washington, USA), all changes were converted to fold-change for consistency.

Meta-analysis could not be carried out due to variation in methods between papers. Differences in cell type, time points used and concentrations of VD used meant that direct comparisons in the form of a meta-analysis was not possible.

### Quality Assessment

The quality assessment method used in this review is a modified version of Risk of Bias (RoB) 2 tool from the Cochrane database to assess risk of bias in randomised trials. This assessment tool has been modified to be appropriate for cell culture experiments such as those included within this review (Supplementary Table 1). Responses in green indicate potential markers for a low risk of bias, orange indicates moderate risk and red indicates potential markers for a high risk of bias (Y = yes, PY = probably yes, PN = probably no, N = no, NI = no information given or not applicable). Questions starting with 1 relate to risk of bias from treatment allocation. Questions starting with 2 relate to risk of bias in measurement of the data. Questions starting with 3 relate to risk of bias in selection of the reported result. Three or four questions were used to assess each section and an overall risk of bias was decided upon. There are three options for overall risk of bias judgement: low risk, high risk or some concerns.

## Results

### Eligibility of Studies

**Figure 2:**
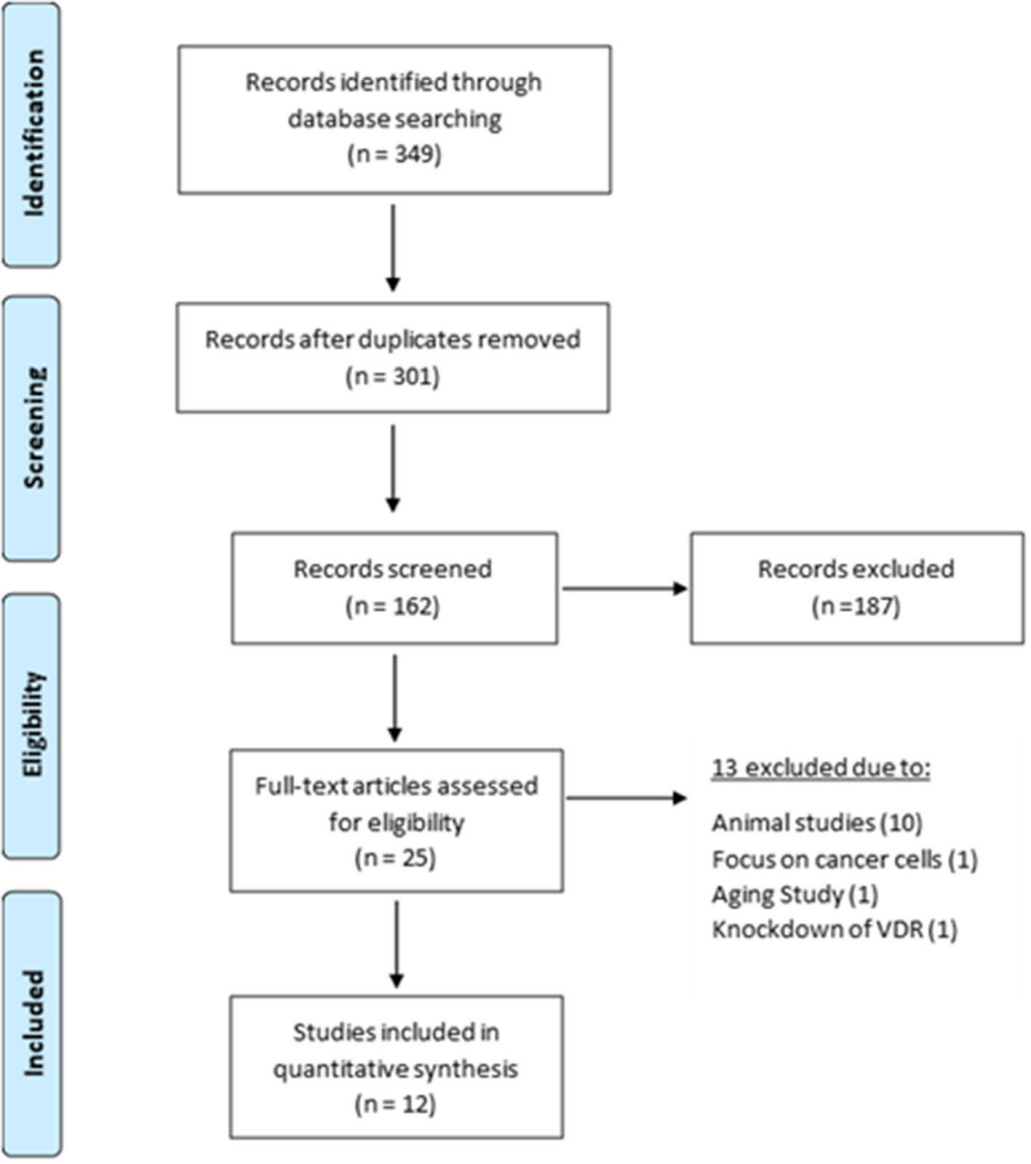
Selection and exclusion of studies in accordance with PRISMA guidelines (Moher et al., 2009).

Using electronic databases (PubMed (MEDLINE), Web of Science and Google Scholar), we identified 349 articles between 1978 and 2020. The removal of duplicates and initial title screen left 301 articles for detailed assessment. Of these 25 were evaluated against the inclusion/exclusion criteria. Ten of these were animal studies and 3 focused on cancer cells, ageing and VD receptor. This left 12 articles eligible for inclusion within this review (Figure 2). A detailed list of excluded studies with reasoning for exclusion can be found in Supplementary Table 2.

### Quality Assessment

All 12 papers received a score of “low risk” when assessed against the quality assessment criteria previously outlined in Supplementary Table 1. For three of the studies (Capiati et al., 1999; Girgis et al., 2014; Olsson et al., 2016) no information could be found regarding replicates and/or repeats therefore it was assumed that this was adequate when giving a low overall bias score (Table 1).

**Table 1:** Summary of quality assessment of included studies.

	**Question**										
**References**	**1.1**	**1.2**	**1.3**	**2.1**	**2.2**	**2.3**	**3.1**	**3.2**	**3.3**	**3.4**	**Rating**
Braga et al. (2017)	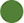	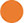	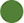	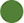	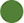	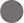	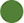	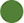	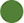	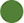	Low
Capiati et al. (1999)	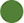	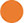	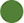	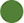	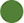	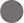	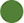	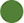	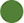	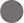	Low
Garcia et al. (2011)	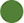	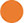	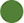	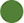	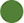	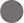	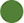	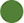	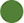	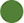	Low
Gili et al. (2016)	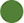	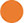	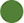	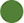	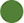	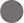	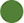	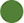	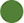	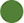	Low
Girgis et al. (2014)	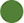	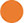	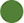	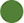	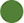	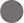	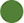	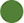	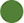	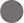	Low
Okuno et al. (2012)	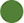	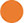	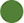	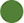	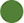	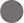	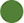	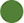	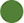	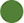	Low
Olsson et al. (2016)	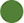	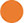	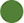	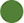	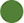	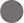	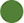	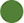	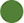	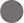	Low
Romeu Montenegro et al. (2019)	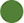	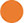	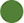	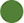	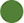	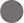	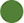	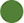	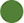	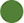	Low
Ryan et al. (2013)	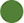	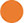	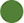	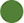	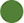	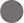	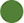	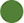	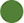	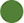	Low
Saini et al. (2019)	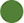	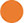	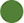	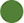	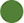	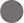	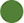	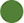	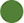	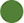	Low
Saito et al. (2017)	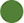	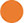	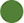	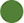	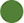	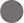	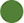	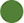	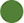	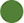	Low
van der Meijden et al. (2016)	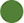	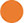	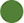	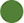	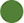	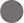	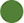	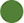	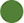	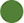	Low

*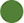 low risk of bias, 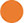 moderate risk of bias, 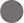 high risk of bias, 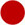 no information given/not applicable*.

### Study Characteristics

All studies included within this review used the biologically active form of VD [1,25(OH)2D3] apart from Saito et al. (2017) where an analogue of the active form of VD called Eldecalcitol was used. Four different cell types were used across the studies (C2C12, primary human myoblasts, primary mouse satellite cells, and primary chick myoblasts) and active VD concentration ranged from 10–5 to 10–13M (Table 2).

**Table 2:** Summary of study characteristics.

**References**	**Cell type**	**Form of vitamin D**	**Concentration**	**Outcomes measured**
Braga et al. (2017)	Mouse skeletal muscle satellite cells	1,25(OH)_2_D_3_	10^−7^M	MyoD, myogenin
Capiati et al. (1999)	Chick myoblasts (obtained from 12-day-old embryo breast tissue)	1,25(OH)_2_D_3_	10^−9^M	Proliferation, creatine kinase, myosin
Garcia et al. (2011)	C2C12	1,25(OH)_2_D_3_	10^−7^M	MyoD, myogenin, myotube size
Gili et al. (2016)	C2C12	1,25(OH)_2_D_3_	10^−9^M	Myogenin, creatine kinase, myosin, myotube size
Girgis et al. (2014)	C2C12	1,25(OH)_2_D_3_	10^−7^M	Myogenin, myotube size
Okuno et al. (2012)	C2C12	1,25(OH)_2_D_3_	10^−7^M, 10^−8^M, and 10^−9^M	Myogenin, myosin
Olsson et al. (2016)	Human skeletal muscle myoblasts	1,25(OH)_2_D_3_	10^−7^M	Proliferation, myoD, myogenin, myosin
Romeu Montenegro et al. (2019)	Human skeletal muscle myoblasts	1,25(OH)_2_D_3_	10^−7^M	Proliferation, myogenin, myosin, myotube size
Ryan et al. (2013)	C2C12	1,25(OH)_2_D_3_	10^−5^M, 10^−7^M, 10^−9^M, 10^−11^M, and 10^−13^M	Myogenin, creatine kinase
Saini et al. (2019)	Human skeletal muscle myoblasts	1,25(OH)_2_D_3_	10^−7^M, 10^−9^M, and 10^−11^M	Proliferation
Saito et al. (2017)	C2C12	Eldecalcitol	10^−7^M, 10^−8^M, and 10^−9^M	MyoD, myosin
van der Meijden et al. (2016)	C2C12	1,25(OH)_2_D_3_	10^−7^M	MyoD, myogenin, myosin, myotube size

### Effects on Proliferation

**Figure 3:**
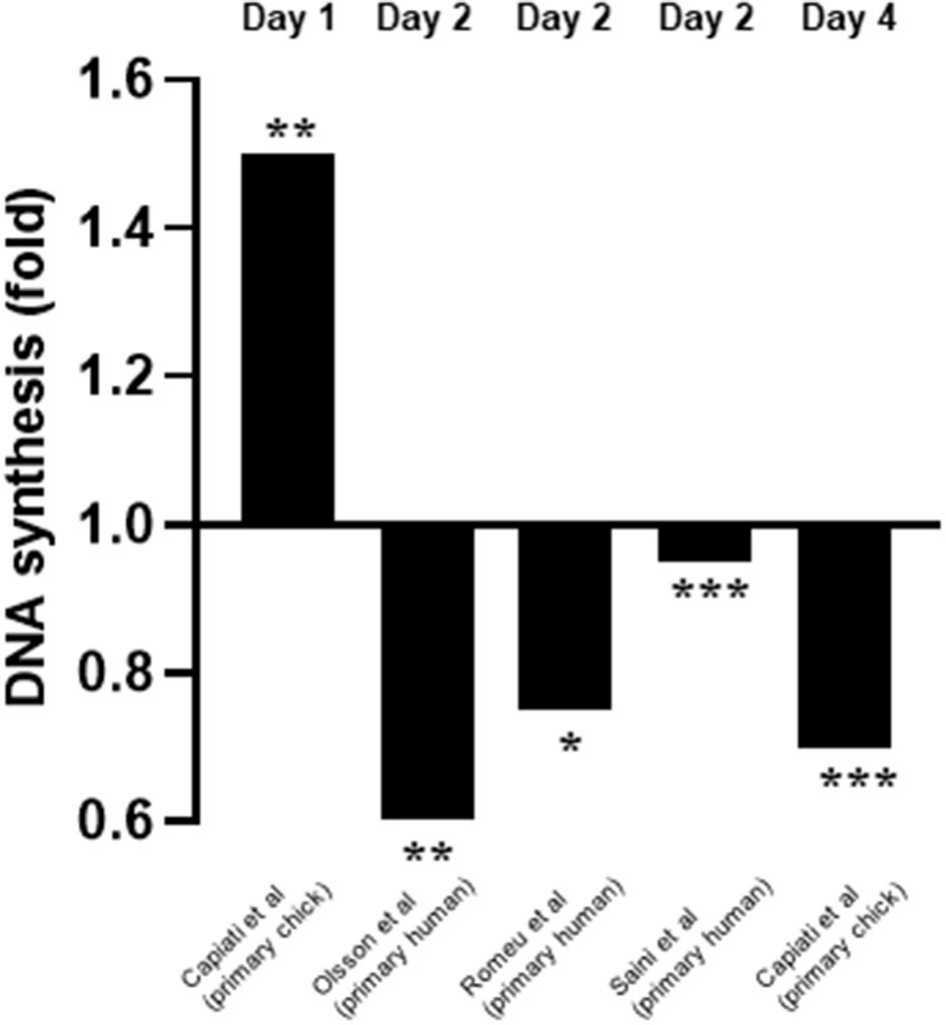
Effect of 1,25(OH)_2_D_3_ on DNA synthesis compared to untreated cells. The active form of vitamin D [1,25(OH)_2_D_3_] at 10^−7^M was used in all studies. *p < 0.05, **p < 0.01, ***p < 0.001.

From the relevant articles, eight (Capiati et al., 1999; Garcia et al., 2011; Okuno et al., 2012; Girgis et al., 2014; Olsson et al., 2016; van der Meijden et al., 2016; Romeu Montenegro et al., 2019; Saini et al., 2019) studied the effects of 1,25(OH)2D3 on proliferation and all reported an inhibitory effect. Of these, four (Capiati et al., 1999; Olsson et al., 2016; Romeu Montenegro et al., 2019; Saini et al., 2019) quantified DNA content as a marker of proliferation (Figure 3). Interestingly, one study (Capiati et al., 1999) reported an initial short stimulatory effect of 1,25(OH)2D3 treatment on DNA synthesis on day 1 (1.5-fold increase) however, this was followed by an inhibitory effect on day 4 (0.7-fold). The remaining three studies (Olsson et al., 2016; Romeu Montenegro et al., 2019; Saini et al., 2019) all revealed a decrease in DNA content of different magnitude (0.5 to 0.95-fold) (Figure 3).

The other four studies measured proliferation in various ways (Table 3). One study showed an increase in p21 and p27 mRNA (Okuno et al., 2012) whilst three studies revealed a decrease in cyclin mRNAs (Girgis et al., 2014; Olsson et al., 2016; Saini et al., 2019). Decreases in proliferation was also shown by an increase in number of cells in the quiescent phase (Okuno et al., 2012; Girgis et al., 2014; Romeu Montenegro et al., 2019), decreased levels of proliferating cell nuclear antigen (PCNA) at the protein level (Garcia et al., 2011) and decreases in DNA synthesis as previously reported. It is important to note that only two out of eight of these studies checked for differences in apoptosis between treated and control cells (Girgis et al., 2014; Olsson et al., 2016).

**Table 3:** Effects of 1,25(OH)_2_D_3_ on proliferation.

**Reference (cell type)**	**VitD form and concentration**	**Effect on proliferation**	**Checked for apoptosis?**
Capiati et al. (1999) (Primary chick)	10^−9^M 1,25(OH)_2_D_3_	[^3^H]thymide incorporation 1.5-fold on day 1, then 0.7-fold on day 4	
Garcia et al. (2011) (C2C12)	10^−7^M 1,25(OH)_2_D_3_	Proliferating cell nuclear antigen (PCNA) protein 0.25-fold on day 7	
Girgis et al. (2014) (C2C12)	10^−7^M 1,25(OH)_2_D_3_	Proliferation 0.4-fold on day 2 23% increase in cells in G_0_/G_1_ quiescent phase on day 2 Cyclin D1 mRNA 0.75-fold on day 2	✓
Okuno et al. (2012) (C2C12)	10^−7^M 1,25(OH)_2_D_3_	17% increase in cells in G_0_/G_1_ quiescent phase on day 3 P21 mRNA 2-fold on day 3 P27 mRNA 3-fold on day 3	
Olsson et al. (2016) (Primary human)	10^−7^M 1,25(OH)_2_D_3_	BrdU incorporation 0.5-fold on day 2 Cyclin D2 mRNA down regulated 3-fold	✓
Romeu Montenegro et al. (2019) (Primary human)	10^−7^M 1,25(OH)_2_D_3_	BrdU incorporation 0.7-fold on day 2 Decrease in number of cells in G_2_/M phase on day 2	
Saini et al. (2019) (Primary human)	10^−7^M 1,25(OH)_2_D_3_	EdU incorporation 0.95-fold on day 2 Down regulation of cyclin A2 and D1 mRNA after 24 h	
van der Meijden et al. (2016) (C2C12)	10^−7^M 1,25(OH)_2_D_3_	27.6% fewer viable cells on day 4	

*All values reported are significant (p < 0.05)*.

### Effects on Differentiation

Differentiation of muscle cells was determined in all but one (Saini et al., 2019) of the final twelve studies. Markers of differentiation included expression of mRNA or protein for myoD (early differentiation), myogenin (early-mid stage), myosin/myosin heavy chain isoforms (late stage), or the measurement of creatine kinase activity (mid-stage). However, it should be noted that the mRNA expression of myogenin and myosin heavy chain isoforms have been shown to change during the time course of differentiation in C2C12 cells (Brown et al., 2012) indicating that the time point at which these markers are measured is important.

### Effects of Vitamin D on Early-Stage Myogenic Differentiation

**Figure 4:**
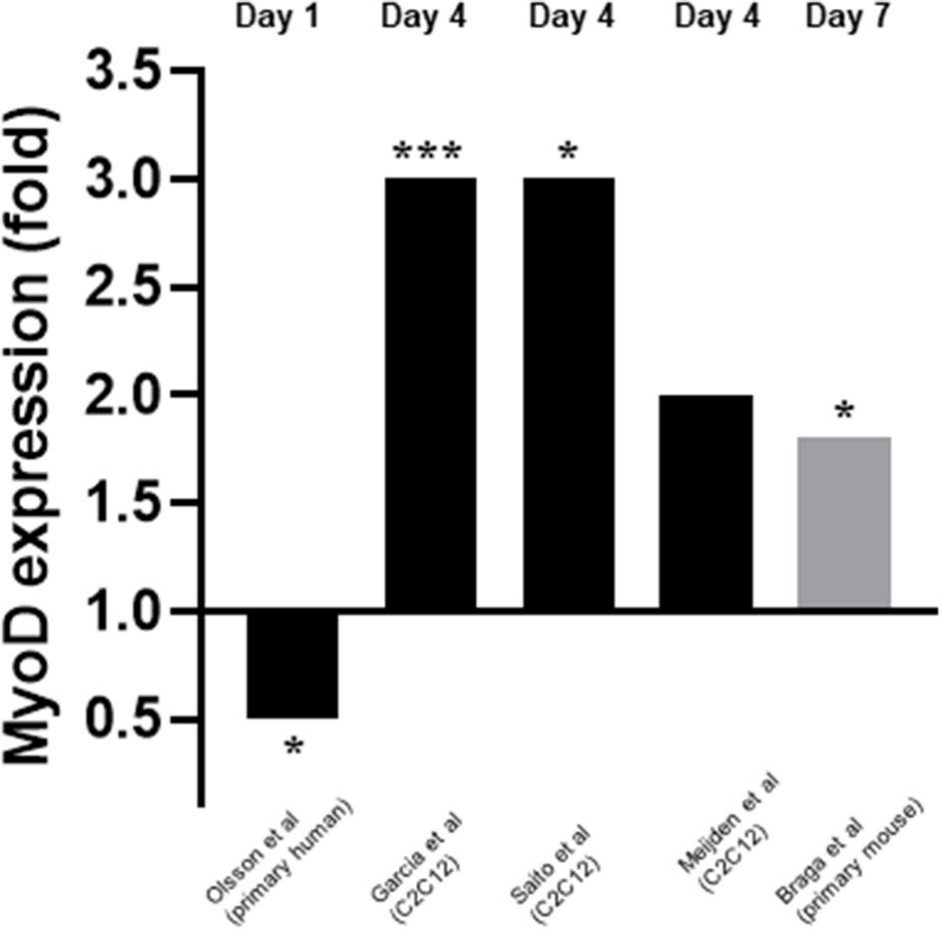
Effect of 1,25(OH)_2_D_3_ or analogue on MyoD mRNA expression. The active form of vitamin D [1,25(OH)_2_D_3_] at 10^−7^M was used in all studies apart from Saito et al. (2017) where Eldecalcitol (an analogue of the active form of vitamin D) was used. Black bars indicate mRNA expression whilst grey indicates protein expression. *p < 0.05, ***p < 0.001.

Five studies measured myoD expression (Garcia et al., 2011; Olsson et al., 2016; van der Meijden et al., 2016; Braga et al., 2017; Saito et al., 2017). Three of these studies measured expression on day 4 (Garcia et al., 2011; van der Meijden et al., 2016; Saito et al., 2017) whilst one measured expression on day 1 (Olsson et al., 2016) and another on day 7 (Braga et al., 2017) (Figure 4). mRNA expression was measured in all cases except for one (Braga et al., 2017) where protein expression was measured. Four out of five studies (Garcia et al., 2011; van der Meijden et al., 2016; Braga et al., 2017; Saito et al., 2017) reported an increase in expression of myoD which ranged from 1.8 to 3-fold. However, one study (Olsson et al., 2016) reported a decrease in expression of 0.5-fold on day 1. These changes in myoD expression were in response to 10–7M 1,25(OH)2D3 for all cases apart from one (Saito et al., 2017) which used 10–7M Eldecalcitol, an analogue of the active form of VD.

### Effects of Vitamin D on Early/Mid-Stage Myogenic Differentiation

**Figure 5:**
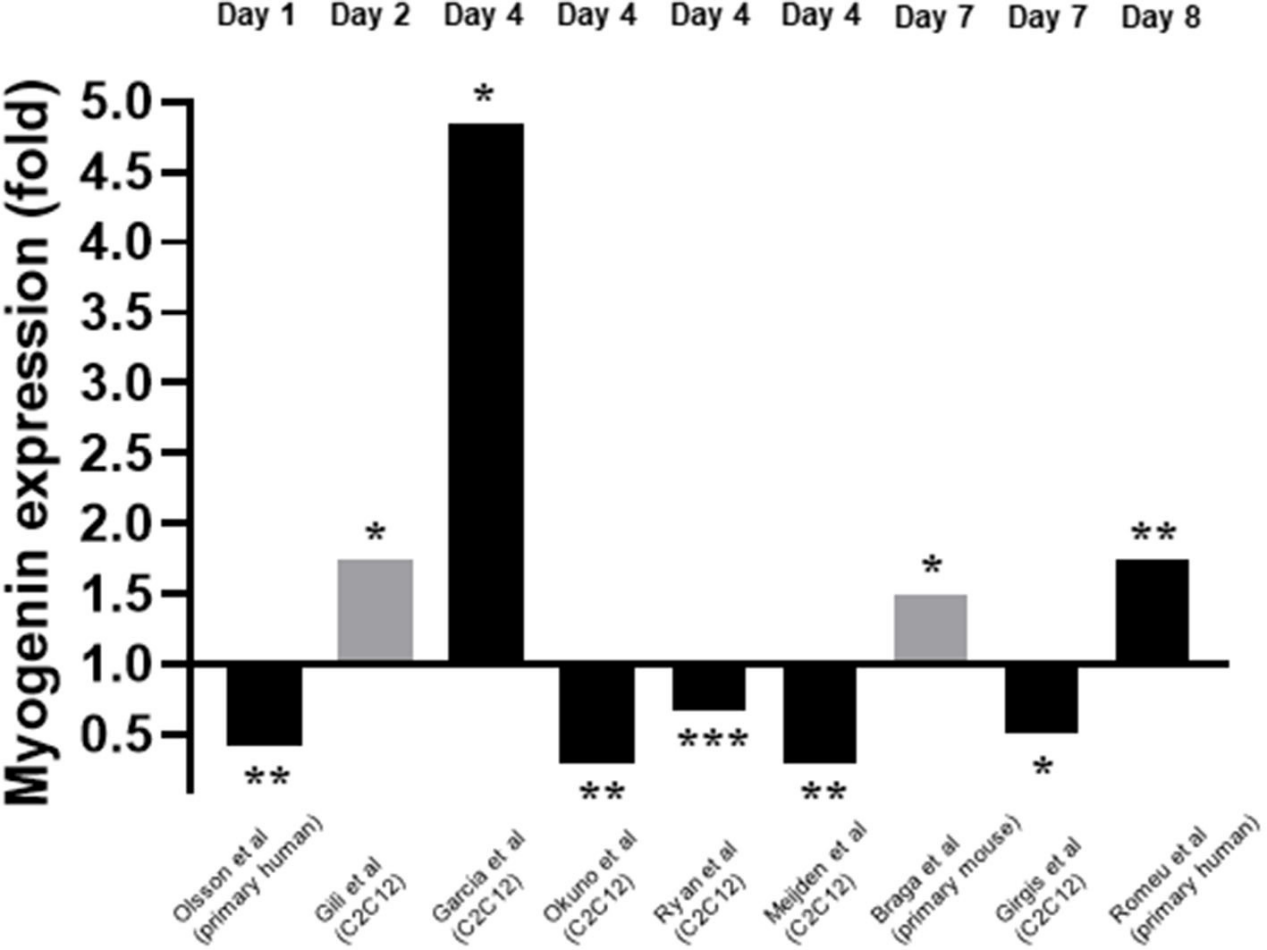
Effect of 1,25(OH)_2_D_3_ of Myogenin expression. The active form of vitamin D [1,25(OH)_2_D_3_] at 10^−7^M was used in all studies apart from Gili et al. (2016) where 10^−8^M was used. Black bars indicate mRNA expression whilst grey indicates protein expression. *p < 0.05, **p < 0.01, ***p < 0.001.

Myogenin expression in response to 1,25(OH)2D3 was investigated by nine of the twelve studies included within this review (Garcia et al., 2011; Okuno et al., 2012; Ryan et al., 2013; Girgis et al., 2014; Gili et al., 2016; Olsson et al., 2016; van der Meijden et al., 2016; Braga et al., 2017; Romeu Montenegro et al., 2019). The time points at which myogenin expression was measured varied from day 1 to day 7. For eight of the nine studies which measured myogenin, the concentration of 1,25(OH)2D3 used was 10–7M but one study (Gili et al., 2016) used 10–8M 1,25(OH)2D3. In most cases mRNA expression was measured but in two studies (Gili et al., 2016; Braga et al., 2017) protein expression was measured. Unlike myoD expression, the level of agreement between studies relating to myogenin expression was low with five studies reporting a decrease in myogenin expression (Okuno et al., 2012; Ryan et al., 2013; Girgis et al., 2014; Olsson et al., 2016; van der Meijden et al., 2016) and four studies reporting an increase in expression (Garcia et al., 2011; Gili et al., 2016; Braga et al., 2017; Romeu Montenegro et al., 2019; Figure 5).

Three studies measured creatine kinase activity as a marker of differentiation (Capiati et al., 1999; Ryan et al., 2013; Gili et al., 2016). Two of these studies reported their results as a time course (Capiati et al., 1999; Gili et al., 2016) whilst one reported results for day 4 only (Ryan et al., 2013). A variety of concentrations of 1,25(OH)2D3 were used across the studies (Table 4). One study (Ryan et al., 2013) reported results for cells grown in either myogenic media or adipogenic media, but only the results for myogenic media have been used to allow comparison to the other studies. Two studies reported an increase in creatine kinase activity which peaked on day 2 following 1,25(OH)2D3 treatment (Capiati et al., 1999; Gili et al., 2016) whilst the other study found that creatine kinase activity decreased across all 1,25(OH)2D3 concentrations on day 4 (Ryan et al., 2013).

**Table 4:** Effects of 1,25(OH)_2_D_3_ on creatine kinase activity.

**Reference** **(Cell type)**	**VitD form and concentration**	**CK activity**	**Significance**
Capiati et al. (1999) (Primary chick)	10^−9^M 1,25(OH)_2_D_3_	−45% on day 1 +55% on day 2 +30% on day 3 + 15% on day 6	*p* < 0.01 *P* < 0.01 *p* < 0.05 *p* < 0.05
Gili et al. (2016) (C2C12)	10^−7^M 1,25(OH)_2_D_3_	1.7-fold on day 1 1.8-fold on day 2 1.3-fold on day 4	Individual *p*-values not given. ANOVA interaction *p* < 0.05
Ryan et al. (2013) (C2C12)	1,25(OH)_2_D_3_ for all: 10^−13^M 10^−11^M 10^−9^M 10^−7^M 10^−5^M	Day 4 for all: Same as control 6% decrease 12.5% decrease 25% decrease 62.5% decrease	Individual *p*-values not given. ANOVA interaction *p* < 0.001

### Effects of Vitamin D on Late-Stage Myogenic Differentiation

A total of seven studies investigated the effects of 1,25(OH)2D3 treatment on myosin protein or mRNA/protein levels of myosin heavy chain (MyHC) isoforms, with the majority measuring the latter (Okuno et al., 2012; Gili et al., 2016; Olsson et al., 2016; van der Meijden et al., 2016; Saito et al., 2017; Romeu Montenegro et al., 2019). MyHC neonatal (MyHC neo) and type IIa (MyHCIIa) were the most commonly studied isoforms across the papers. Time points of expression varied greatly between studies. In some cases, expression was measured as early as day 1 whereas others measured up to day 8 (Table 5). Overall, one study found an increase in myosin protein (Capiati et al., 1999), one study found and increase in MyHC protein (Gili et al., 2016), three studies reported an increase in expression of at least one MyHC isoform (Okuno et al., 2012; Gili et al., 2016; van der Meijden et al., 2016; Saito et al., 2017) and two studies reported a decrease in MyHC isoforms (MyHC neo, MyHC IIa and MyHCII subtype unspecified) (Olsson et al., 2016; Romeu Montenegro et al., 2019).

**Table 5:** Effects of 1,25(OH)_2_D_3_ or eldecalcitol on myosin or myosin heavy chain isoform expression.

**Reference (Cell type)**	**VitD concentration and form**	**Factor measured**	**Effect**	**Significance**
Capiati et al. (1999) (Primary chick)	10^−9^M 1,25(OH)_2_D_3_	Myosin protein	+88% on day 2 +31.5% on day 6	*p* < 0.01 *p* < 0.01
Gili et al. (2016) (C2C12)	10^−9^M 1,25(OH)_2_D_3_	MyHC protein	1.2-fold on day 2 1.4-fold on day 4	*p*-values not given
Okuno et al. (2012) (C2C12)	10^−7^M 1,25(OH)_2_D_3_	MyHC neo mRNA MyHCIIa mRNA	0.4-fold on day 4 2.5-fold on day 8	*p* < 0.05 *p* < 0.01
Olsson et al. (2016) (Primary human)	10^−7^M 1,25(OH)_2_D_3_	MyHC neo mRNA MyHCIIa mRNA	0.66-fold on day 1 0.73-fold on day 1	No *p*-values given
Romeu Montenegro et al. (2019) (Primary human)	10^−7^M 1,25(OH)_2_D_3_	MyHCII mRNA	0.4-fold on day 5	*p* < 0.01
Saito et al. (2017) (C2C12)	10^−8^M eldecalcitol	MyHC neo mRNA MyHCIIa mRNA	1.4-fold on day 4 1.8-fold on day 4	Not significant *p* < 0.01
van der Meijden et al. (2016) (C2C12)	10^−7^M 1,25(OH)_2_D_3_	MyHCIIa mRNA	2.5-fold on day 3	*p*-value not given

*1,25(OH)2D3, 1,25 dihydroxyvitamin D; MyHC, myosin heavy chain*.

**Figure 6:**
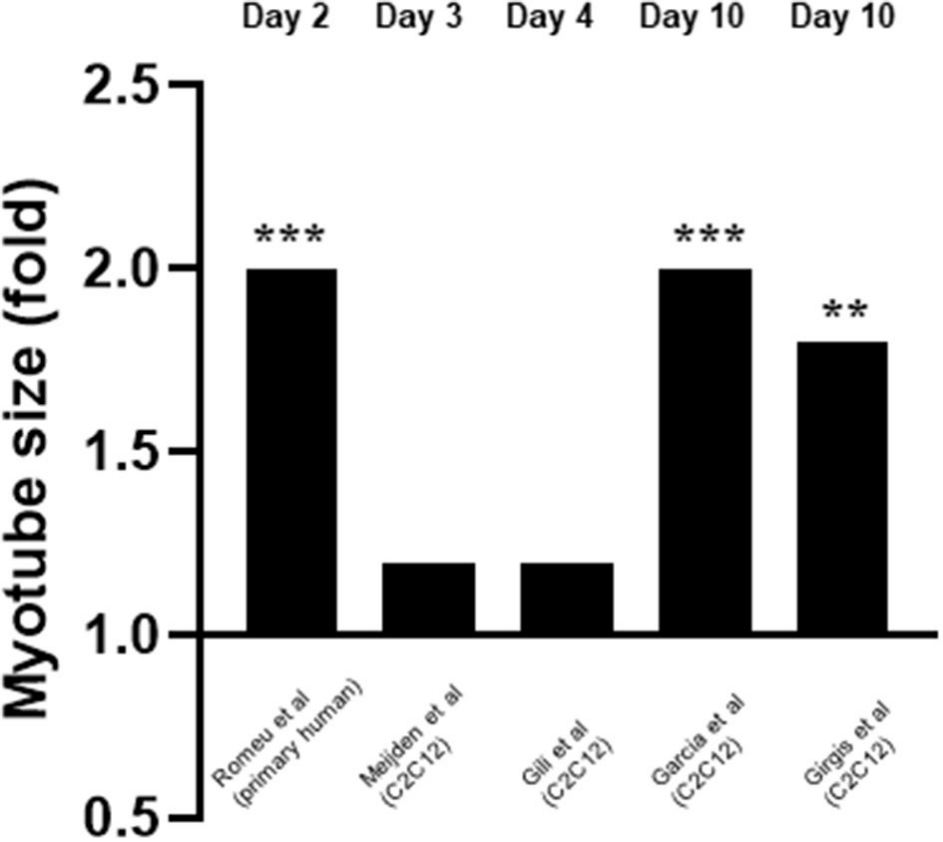
Effect of 1,25(OH)_2_D_3_ on myotube size. 5 studies investigated the effect of 1,25(OH)_2_D_3_ on myotube size. Myotube size was measured at varying time points which ranged from day 3 to day 10. 1,25(OH)_2_D_3_ concentration was 10^−7^M for all cases apart from Gili et al. (2016) where 10^−9^M was used. **p < 0.01, ***p < 0.001.

Five studies measured the effects of 1,25(OH)2D3 on myotube size (Garcia et al., 2011; Girgis et al., 2014; Gili et al., 2016; van der Meijden et al., 2016; Romeu Montenegro et al., 2019; Figure 6). This was also measured at varying time points from day 2 to day 10. For one study 10–9M 1,25(OH)2D3 was used (Gili et al., 2016) whilst 10–7M 1,25(OH)2D3 was used in the other four (Garcia et al., 2011; Girgis et al., 2014; van der Meijden et al., 2016; Romeu Montenegro et al., 2019). All five studies concluded that treatment with 1,25(OH)2D3 resulted in an increase in myotube size which ranged from 1.1 to 2-fold.

## Discussion

This review has shown good agreement across the different studies in terms of the active form of VD inhibiting muscle cell proliferation. However, the effects on differentiation, as determined by various markers, showed less consistency, probably due to a combination of different cell types and time points being used.

### Treatment With Vitamin D Inhibits Proliferation

Of the eight studies which investigated the effects of active VD on muscle cell proliferation (Capiati et al., 1999; Garcia et al., 2011; Okuno et al., 2012; Girgis et al., 2014; Olsson et al., 2016; van der Meijden et al., 2016; Romeu Montenegro et al., 2019; Saini et al., 2019), all of them found an inhibitory effect. One study observed a stimulatory effect on day 1, however this was followed by inhibition on day 4 (Capiati et al., 1999). It is worth noting that only two studies (Girgis et al., 2014; Olsson et al., 2016) checked for differences in apoptosis between treated and untreated groups therefore it cannot be ruled out that the decrease in DNA observed in some of the other studies was not due to apoptosis.

Importantly, Okuno et al. (2012) showed that active VD caused an increase in cell cycle arrest at G0/G1 which occurred in parallel with increased expression of p21 and p27. Both p21 and p27 are members of the Cip/Kip family and are able to bind to cyclin dependent kinases and inhibit their role in cell cycle progression (Bachs et al., 2018). In order for a cell to proliferate, expression of p21 must decrease to a level where it no longer forms a complex with p53 (Terzi et al., 2016). Additionally, cells must also reduce/eliminate p27 to progress through proliferation, which is achieved via translocation of p27 to the cytoplasm where it is degraded (Bachs et al., 2018). Both Okuno et al. (2012) and Olsson et al. (2016) reported increased expression of both p21 and p27 suggesting that active VD treatment leads to increased transcription of these factors which likely contributes to the inhibition of cell proliferation.

Two studies reported that active VD decreased expression of both cyclin A2 and cyclin D3 (Olsson et al., 2016; Saini et al., 2019). Cyclin A2 is able to bind to and activate two cyclin dependent kinases (CDKs) required for cell cycle progression: CDK4 as DNA synthesis begins during S phase and CDK1 during the transition from G2 to M phase (Pagano et al., 1992). The D cyclins activate CDK4/6 enabling entry into S phase and down-regulation of cyclin D3 specifically inhibits G1 to S transition (Bartkova et al., 1998). From this, it can be suggested that active VD represses transcription of at least two cyclins which leads to inhibition of cell cycle transition and therefore cell cycle arrest and inhibition of proliferation.

**Figure 7:**
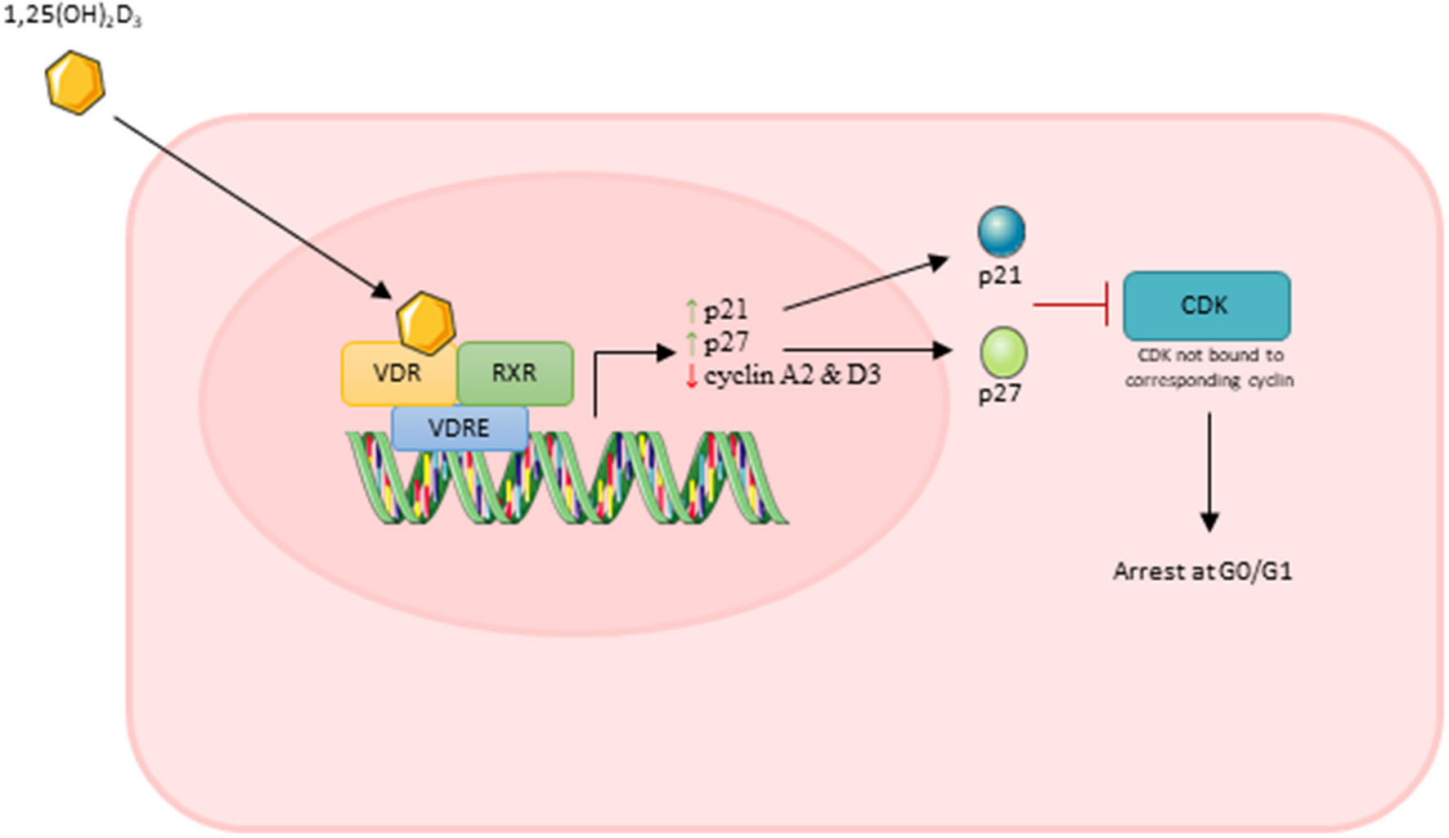
Effect of active vitamin D [1,25(OH)_2_D_3_] on myoblast proliferation. Images used within this figure were obtained from smart servier medical art and can be found at https://smart.servier.com.

Overall, decreases in DNA synthesis, increases in expression of p21/p27 and decreases in expression of cyclin A2/D3 suggest that treatment with active VD has a strong anti-proliferative effect on muscle cells in culture. It is likely that the cumulative effect of all of these factors lead to an overall reduction in muscle cell proliferation. The process of this anti-proliferative effect of active VD is shown in Figure 7.

### Vitamin D Appears to Stimulate Early-Stage Differentiation

**Figure 8:**
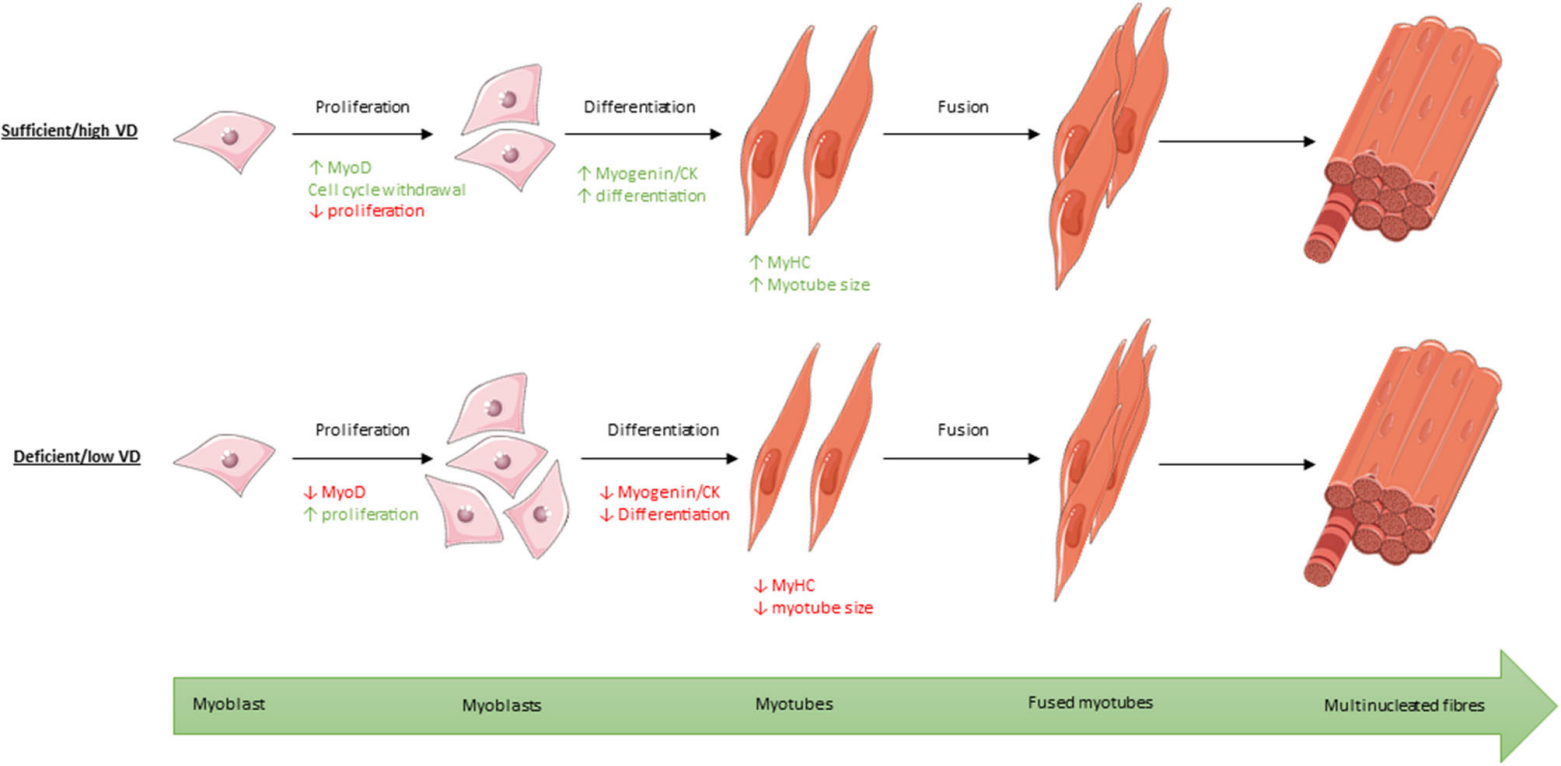
Process of myogenic differentiation from myoblasts to multinucleated muscle fibres showing the effect of high/sufficient active vitamin D (VD) on various transcription factors within the process compared to low/deficient levels. Images used within this figure were obtained from smart servier medical art and can be found at https://smart.servier.com.

Myogenesis is a highly ordered and sequential process, guided by several transcription factors at various stages. This process of myogenic differentiation, and the proposed effect of active vitamin D on this process, is shown in Figure 8. Following withdrawal from the cell cycle, as described previously, myoblast fusion occurs to form multinucleated myotubes (Garcia et al., 2011). MyoD is a transcription factor involved in the early stages of differentiation (Girgis et al., 2014). When subjected to culture conditions which should induce differentiation, myoD^−/−^ cells have been shown to continue to proliferate suggesting that expression of myoD is essential for withdrawal from the cell cycle (Sabourin et al., 1999). However, we previously observed no change in myoD mRNA over the time course of differentiation in C2C12 cells (Brown et al., 2012).

Four of the five studies which measured myoD reported an increase in expression following treatment with active VD (Garcia et al., 2011; van der Meijden et al., 2016; Braga et al., 2017; Saito et al., 2017), although the increase in expression was not significant for one study (van der Meijden et al., 2016). Interestingly, the only study which found a decrease in myoD expression was also the only study which used primary human cells (Olsson et al., 2016). This suggests that the effects of active VD on differentiation may depend upon cell type and/or species. However, this study also measured myoD very early in the process (day 1) (Olsson et al., 2016) whereas the remaining four studies all reported increased myoD expression on either day 4 (Garcia et al., 2011; van der Meijden et al., 2016; Saito et al., 2017) or day 7 (Braga et al., 2017). The effects observed may depend on upon the cell type and/or time point.

Evidence has shown that inhibition of IGFII results in a decrease in expression of myoD target genes, suggesting that IGFII is a key regulator of myoD expression (Wilson and Rotwein, 2006). One of the studies which found a 1.8-fold increase in myoD expression (Braga et al., 2017) found that both IGFI and IGFII expression also increased at the same time point. Additionally, increased expression of the IGFs has been shown to inhibit Myostatin, the only known negative regulator of muscle mass (Retamales et al., 2015). These findings suggest that active VD may increase expression of myoD directly or possibly indirectly via effects on local expression of IGFI and/or IGFII.

### Effects of Vitamin D on Mid-Stage Differentiation Are Cell Type and Time Dependent

Induction of myogenin expression precedes the fusion of myoblasts to form myotubes, then myogenin switches on transcription of various muscle-specific genes (e.g., creatine kinase and MyHC isoforms) expressed by myotubes and muscle fibres (Bentzinger et al., 2012). Myogenin expression is therefore used as a marker of early to mid-stage differentiation and normally follows an increase in myoD expression (Hernández-Hernández et al., 2017). Indeed, myogenin knockout mice die immediately following birth, and whilst they have myoblasts present within the muscle, no muscle fibres are formed, resulting in the complete absence of functional skeletal muscle (Hernández-Hernández et al., 2017). Importantly, myogenin expression is completely blocked when the VD receptor is knocked down *in vitro* suggesting that VD has a direct effect on myogenin expression via the VDR (Gili et al., 2016).

However, the nature of this effect is controversial. As seen in Figure 5, five studies reported a decrease in myogenin expression (Okuno et al., 2012; Ryan et al., 2013; Girgis et al., 2014; Olsson et al., 2016; van der Meijden et al., 2016) whilst four reported an increase (Garcia et al., 2011; Gili et al., 2016; Braga et al., 2017; Romeu Montenegro et al., 2019). One possible explanation for this is the difference in methods between studies. Differentiation can be triggered via two mechanisms *in vitro*: 1. Serum starvation which leads to a decrease in mitogenic stimuli, withdrawal from the cell cycle and increase in myogenin expression. 2. Prolonged confluence leading to a high cell density and more cell-cell contacts, which leads to increased IGF expression and an increase in myogenin (Girgis et al., 2014). Garcia et al. (2011), who used the latter method, found that myogenin expression was increased at day 4 following active VD treatment of C2C12 cells. On the other hand, Girgis et al. (2014) used the serum deprivation method and reported a decrease in myogenin expression in C2C12 cells at day 7. It is important to note that we previously showed (Brown et al., 2012; Brearley et al., 2019) using multiple time points, that myogenin mRNA initially increases upon induction of differentiation (via serum starvation) of C2C12 cells, reaching a peak around day 2-3, then decreases again. Therefore, induction of differentiation would be associated with an increase in myogenin mRNA at early time points (days 0–3), but increased differentiation could also be associated with a more rapid decline in expression at later time points. It is also worth noting that C2C12 cells differentiate more rapidly than primary human myoblasts (Cheng et al., 2014) so are likely to have an earlier peak in myogenin expression. Hence, the discrepancies in the observed effects of active VD on myogenin expression could be due to the cell type used, the timepoints of measurement or a combination of the two. It is important that future studies should include measurements at several time points in order to make clear interpretations. It is also plausible that the two different methods of inducing differentiation may have different time frames, such that the rates of increase and decrease in expression as well as the peak of myogenin may be different. Certainly, there were large differences between studies in time points at which myogenin expression was measured, which likely contributed to the conflicting results.

Creatine kinase (CK) is a mid-stage marker of differentiation reported to peak around day 4 to 6 in both C2C12 (Brown et al., 2012) and primary chick myoblasts (Capiati et al., 1999). Two studies (Capiati et al., 1999; Gili et al., 2016) reported an increase in CK activity following treatment with active VD both of which found expression to peak on day 2, earlier than the expected window of 4–6 days. The remaining study which looked at CK activity (Ryan et al., 2013) reported a decrease in activity in C2C12 cells on day 4 across all active VD concentrations studied. Once again this might relate to differences in the timing relative to the expected peak in expression, with an early increase and a later decrease in expression potentially indicating an increase in the rate of differentiation.

Overall, the data is conflicting for both markers of early to mid-stage differentiation (myogenin and creatine kinase), but this may be due to the varying time points that each marker was measured, the variation in cell type, the differing concentrations of active VD used or a combination of all three.

### Vitamin D Stimulates Expression of Late-Stage Markers of Differentiation

Myosin and the myosin heavy chain (MyHC) isoforms are muscle specific proteins that are often used as markers of mature, differentiated muscle cells (Gili et al., 2016) and together they form a significant proportion of the proteins present in differentiated muscle (Zammit, 2017). Five studies reported an increase in myosin or MyHC isoforms following active VD treatment (Capiati et al., 1999; Okuno et al., 2012; Gili et al., 2016; van der Meijden et al., 2016; Saito et al., 2017) whilst two studies reported a decrease in expression (Olsson et al., 2016; Romeu Montenegro et al., 2019). We previously showed that the MyHC isoforms are expressed in two distinct patterns during differentiation in C2C12 cells (Brown et al., 2012). The first pattern is an increase then decrease, peaking around day 2–4 of mRNA for MyHC embryonic (MyHC emb), foetal (MyHC neo), and slow type 1 (MyHC I) isoforms (Brown et al., 2012). The fast type II isoforms were all expressed much later in differentiation, being induced at days 2–4 in the order IIa > IIx > IIb (Brown et al., 2012). Hence, an increase in differentiation would always results in an increase in expression of the fast (type II) isoforms, but effects on the embryonic, foetal, and slow (type I) isoforms would be time dependent, with an increase in expression at early time points, but a decrease in expression at later time points.

Okuno et al. (2012) found that MyHC type IIa expression in C2C12 cells was increased by active VD at day 8 and they suggest that this indicated an anabolic effect in muscle. On the other hand, Olsson et al. (2016) found that both MyHC neonatal and MyHC IIa expression were reduced in C2C12 cells at day 1. Considering MyHC is a marker of late stage differentiation (Gili et al., 2016) it is unclear why this study chose to measure MyHC expression during the earlier stages of differentiation, possibly missing the timepoint where MyHC expression may have increased.

The majority of *in vitro* evidence suggests that active VD stimulates the expression of MyHC isoforms, suggesting that active VD stimulates differentiation. Additionally, one study showed that injection of active VD increased expression of MyHC type IIa *in vivo* (Korn et al., 2013). However, this could be due to effects on muscle fibre type rather than muscle cell differentiation. It is known that IGFI can alter MyHC isoform expression (Saito et al., 2017) so active VD may impact on muscle fibre type indirectly via induction of local IGFI expression. Supporting this, Braga et al. (2017) reported an increase in expression of both IGFI and IGFII following active VD treatment in primary mouse cells *in vitro*. However, some argue that non-genomic actions of active VD, such as increases in intracellular Calcium concentrations, may be responsible for its effects on MyHC mRNA expression (de Boland and Boland, 1987).

### Vitamin D Increases Myotube Size

Of the five studies which measured myotube size, all five reported an increase in myotube size (Garcia et al., 2011; Girgis et al., 2014; Gili et al., 2016; van der Meijden et al., 2016; Romeu Montenegro et al., 2019), which suggests a stimulatory effect of active VD on differentiation. In addition, Garcia et al. (2011) found an increase in expression of Follistatin (Fst). Fst is an antagonist of Myostatin (Mstn) a known negative regulator of muscle mass (Retamales et al., 2015), including both muscle cell proliferation and differentiation. Therefore, active VD might increase myotube size directly and/or indirectly via increasing IFG1 or Fst expression, the latter then inhibits Mstn (an inhibitor of differentiation) but both result in increased differentiation. Supporting this, Girgis et al. (2014) found myotube size was increased 1.8-fold on day 10 following a 10-fold decrease in Myostatin expression on day 7.

## Conclusions

There is reasonably strong evidence to suggest that active VD inhibits proliferation of myoblasts, and stimulates differentiation and increases myotube size, although the effects on each stage of differentiation are not entirely consistent. These inconsistencies may relate to the use of different cell types and measurements at variable time points which makes interpretation more difficult. However, understanding the normal time course of expression during differentiation allows for some consistency across studies, but it clearly indicates that future studies should involve multiple time points. Also, only one study (Ryan et al., 2013) used concentrations of active VD within the physiological serum range (around 10–10 M) (Hou et al., 2018) so future studies should also consider using concentrations of active VD which are more physiologically relevant. However, it is worth noting that muscle cells do express 1α-hydroxylase and therefore can locally convert inactive VD to the active form (Mori et al., 2020). As it is not possible to measure these transient, local fluctuations in active VD, it cannot be ruled out that it may be possible for intracellular physiological concentrations to reach levels used within some of the studies in this review (10–7 M).

Due to the presence of 1α-hydroxylase within skeletal muscle (Mori et al., 2020) future studies should also investigate the effects of inactive VD on muscle cells to see whether this results in similar effects to the active form.

It does appear that active VD has effects on skeletal muscle, particularly muscle cell proliferation and differentiation, indicating potential effects during embryonic development; when these processes mainly take place. VD deficiency has been shown to increase the risk of poor muscle strength and therefore falls, particularly in the elderly population (Garcia et al., 2011; Ceglia and Harris, 2013), but this review suggests that VD deficiency during embryonic and foetal development (i.e., during pregnancy) may also impact upon muscle development and function. Whilst VD supplementation in deficient individuals appears effective in increasing muscle strength and therefore decreasing fall risk in the elderly (Ryan et al., 2013), more research is needed to determine the impacts of supplementation during pregnancy/lactation or in the young offspring on muscle cell differentiation.

## Data Availability Statement

The original contributions presented in the study are included in the article/Supplementary Material, further inquiries can be directed to the corresponding author/s.

## Author Contributions

KA and JB contributed to the conception and interpretation of the data and reviewing of the draft manuscript. KA contributed to writing the original draft manuscript, acquisition, and analysis of the data. SK contributed to the data acquisition. TP and PJ contributed to the revising and contributing intellectual content writing. JB had final approval of the version to be published. All authors contributed to the article and approved the submitted version.

## Funding

The work was supported by the BBSRC-DTP studentships to KA and SK (Grant No. BB/M008770/1).

## Conflict of Interest

The authors declare that the research was conducted in the absence of any commercial or financial relationships that could be construed as a potential conflict of interest.

## Publisher's Note

All claims expressed in this article are solely those of the authors and do not necessarily represent those of their affiliated organizations, or those of the publisher, the editors and the reviewers. Any product that may be evaluated in this article, or claim that may be made by its manufacturer, is not guaranteed or endorsed by the publisher.
