# Supplementary material for: In vitro Effects of Biologically Active Vitamin D on Myogenesis: A Systematic Review
Source: Front Physiol. 2021 Sep 9;12:736708. doi: 10.3389/fphys.2021.736708 (PMC8458760; doi:10.3389/fphys.2021.736708)
Supplement: Supplementary file 1 [file Table_1.docx]

**Supplementary Material**

**Supplementary Table 1**. Quality Assessment tool to assess risk of bias to treatment allocation (1), risk of measurement bias (2) and risk of reported result Bias (3).

| **Signalling questions** | **Comments** | **Response options** |
| --- | --- | --- |
| 1.1 Was allocation to control/treated random? |  | **Y / PY / PN / N / NI** |
| 1.2 Was the researcher aware of which cells were treated or control |  | **Y / PY / PN / N / NI** |
| 1.3 Were cells in the same conditions before treatment allocation? |  | **Y / PY / PN / N / NI** |
| **Risk of treatment allocation bias judgement** | | |
| 2.1 Was the method measuring the outcome appropriate? |  | **Y / PY / PN / N / NI** |
| 2.2 Were there any differences in measurement between treatment groups? |  | **Y / PY / PN / N / NI** |
| 2.3 If yes to 2.2 could this have been due to knowledge of expected effects? |  | **Y / PY / PN / N / NI** |
| **Risk of measurement bias judgement** | | |
| 3.1 Was data analysed in accordance with a pre-specified analysis plan? |  | **Y / PY / PN / N / NI** |
| 3.2 Are any results likely to have been selected from… multiple eligible outcome measurements? |  | **Y / PY / PN / N / NI** |
| 3.3 …Multiple eligible analyses of the data? |  | **Y / PY / PN / N / NI** |
| 3.4 Were adequate repeats of each experiment carried out? |  | **Y / PY / PN / N / NI** |
| **Risk of selection of result bias judgement** | | |
| **Key:** Y = yes, PY = probably yes, PN = probably no, N = no, NI = no information given or not applicable. | | |
