# Supplementary material for: In vitro Effects of Biologically Active Vitamin D on Myogenesis: A Systematic Review
Source: Front Physiol. 2021 Sep 9;12:736708. doi: 10.3389/fphys.2021.736708 (PMC8458760; doi:10.3389/fphys.2021.736708)
Supplement: Supplementary file 2 [file Table_2.docx]

**Supplementary Table 2.** List of excluded studies and reason for exclusion.

| **Reference** | **Reason for exclusion** |
| --- | --- |
| [1] | This study looked at the effects of vitamin D on smooth muscle cells. Due to the focus of the review being skeletal muscle cells, this review was excluded. |
| [2] | This study administered vitamin D to live rats, so vitamin D treatment was *in vivo.* This was not in line with the inclusion criteria of *in vitro* studies. |
| [3] | This study administered vitamin D to sea bass larvae, so vitamin D treatment was *in vivo.* This was not in line with the inclusion criteria of *in vitro* studies. |
| [4] | This study administered vitamin D to live C57BL/6 J mice, so vitamin D treatment was *in vivo.* This was not in line with the inclusion criteria of *in vitro* studies. |
| [5] | Muscle biopsies taken from cancer patients making it difficult to compare results to muscle cells from healthy patients/cell lines. |
| [6] | This study administered vitamin D to live wistar rats, so vitamin D treatment was *in vivo.* This was not in line with the inclusion criteria of *in vitro* studies. |
| [7] | This study administered vitamin D to live mice, so vitamin D treatment was *in vivo.* This was not in line with the inclusion criteria of *in vitro* studies. The VDR was also knocked down so was not comparable to other studies. |
| [8] | This study looked at the effects of vitamin D on cardiomyocyte cells. Due to the focus of the review being skeletal muscle cells, this review was excluded. |
| [9] | This study administered vitamin D to live broiler chickens, so vitamin D treatment was *in vivo.* This was not in line with the inclusion criteria of *in vitro* studies. |
| [10] | This study used an *in vitro* model using C2C12 cells however, they knocked down the VDR using shRNA which makes it difficult to compare to the other studies within the review. |
| [11] | This study administered vitamin D to live rats, so vitamin D treatment was *in vivo.* This was not in line with the inclusion criteria of *in vitro* studies. |
| [12] | This study administered vitamin D to live rats, so vitamin D treatment was *in vivo.* This was not in line with the inclusion criteria of *in vitro* studies. |
| [13] | This study administered vitamin D to live rats, so vitamin D treatment was *in vivo.* This was not in line with the inclusion criteria of *in vitro* studies. |

Reference list of excluded studies

1. Abe, H.; Iehara, N.; Utsunomiya, K.; Kita, T.; Doi, T. A vitamin D analog regulates mesangial cell smooth muscle phenotypes in a transforming growth factor-beta type II receptor-mediated manner. *J Biol Chem* **1999**, *274*, 20874-20878, doi:10.1074/jbc.274.30.20874.

2. Akagawa, M.; Miyakoshi, N.; Kasukawa, Y.; Ono, Y.; Yuasa, Y.; Nagahata, I.; Sato, C.; Tsuchie, H.; Nagasawa, H.; Hongo, M., et al. Effects of activated vitamin D, alfacalcidol, and low-intensity aerobic exercise on osteopenia and muscle atrophy in type 2 diabetes mellitus model rats. *PLoS One* **2018**, *13*, e0204857, doi:10.1371/journal.pone.0204857.

3. Alami-Durante, H.; Cluzeaud, M.; Bazin, D.; Mazurais, D.; Zambonino-Infante, J.L. Dietary cholecalciferol regulates the recruitment and growth of skeletal muscle fibers and the expressions of myogenic regulatory factors and the myosin heavy chain in European sea bass larvae. *J Nutr* **2011**, *141*, 2146-2151, doi:10.3945/jn.111.146118.

4. Belenchia, A.M.; Jones, K.L.; Will, M.; Beversdorf, D.Q.; Vieira-Potter, V.; Rosenfeld, C.S.; Peterson, C.A. Maternal vitamin D deficiency during pregnancy affects expression of adipogenic-regulating genes peroxisome proliferator-activated receptor gamma (PPARgamma) and vitamin D receptor (VDR) in lean male mice offspring. *Eur J Nutr* **2018**, *57*, 723-730, doi:10.1007/s00394-016-1359-x.

5. Camperi, A.; Pin, F.; Costamagna, D.; Penna, F.; Menduina, M.L.; Aversa, Z.; Zimmers, T.; Verzaro, R.; Fittipaldi, R.; Caretti, G., et al. Vitamin D and VDR in cancer cachexia and muscle regeneration. *Oncotarget* **2017**, *8*, 21778-21793, doi:10.18632/oncotarget.15583.

6. Domingues-Faria, C.; Chanet, A.; Salles, J.; Berry, A.; Giraudet, C.; Patrac, V.; Denis, P.; Bouton, K.; Goncalves-Mendes, N.; Vasson, M.P., et al. Vitamin D deficiency down-regulates Notch pathway contributing to skeletal muscle atrophy in old wistar rats. *Nutr Metab (Lond)* **2014**, *11*, 47, doi:10.1186/1743-7075-11-47.

7. Endo, I.; Inoue, D.; Mitsui, T.; Umaki, Y.; Akaike, M.; Yoshizawa, T.; Kato, S.; Matsumoto, T. Deletion of vitamin D receptor gene in mice results in abnormal skeletal muscle development with deregulated expression of myoregulatory transcription factors. *Endocrinology* **2003**, *144*, 5138-5144, doi:10.1210/en.2003-0502.

8. Hlaing, S.M.; Garcia, L.A.; Contreras, J.R.; Norris, K.C.; Ferrini, M.G.; Artaza, J.N. 1,25-Vitamin D3 promotes cardiac differentiation through modulation of the WNT signaling pathway. *J Mol Endocrinol* **2014**, *53*, 303-317, doi:10.1530/JME-14-0168.

9. Hutton, K.C.; Vaughn, M.A.; Litta, G.; Turner, B.J.; Starkey, J.D. Effect of vitamin D status improvement with 25-hydroxycholecalciferol on skeletal muscle growth characteristics and satellite cell activity in broiler chickens. *J Anim Sci* **2014**, *92*, 3291-3299, doi:10.2527/jas.2013-7193.

10. Irazoqui, A.P.; Boland, R.L.; Buitrago, C.G. Actions of 1,25(OH)2-vitamin D3 on the cellular cycle depend on VDR and p38 MAPK in skeletal muscle cells. *J Mol Endocrinol* **2014**, *53*, 331-343, doi:10.1530/JME-14-0102.

11. Kinoshita, H.; Miyakoshi, N.; Kasukawa, Y.; Sakai, S.; Shiraishi, A.; Segawa, T.; Ohuchi, K.; Fujii, M.; Sato, C.; Shimada, Y. Effects of eldecalcitol on bone and skeletal muscles in glucocorticoid-treated rats. *J Bone Miner Metab* **2016**, *34*, 171-178, doi:10.1007/s00774-015-0664-4.

12. Max, D.; Brandsch, C.; Schumann, S.; Kühne, H.; Frommhagen, M.; Schutkowski, A.; Hirche, F.; Staege, M.S.; Stangl, G.I. Maternal vitamin D deficiency causes smaller muscle fibers and altered transcript levels of genes involved in protein degradation, myogenesis, and cytoskeleton organization in the newborn rat. *Mol Nutr Food Res* **2014**, *58*, 343-352, doi:10.1002/mnfr.201300360.

13. Oku, Y.; Tanabe, R.; Nakaoka, K.; Yamada, A.; Noda, S.; Hoshino, A.; Haraikawa, M.; Goseki-Sone, M. Influences of dietary vitamin D restriction on bone strength, body composition and muscle in rats fed a high-fat diet: involvement of mRNA expression of MyoD in skeletal muscle. *J Nutr Biochem* **2016**, *32*, 85-90, doi:10.1016/j.jnutbio.2016.01.013.
